# Supplementary figures and images for: Towards triple elimination of HIV, syphilis and HBV mother-to-child transmission: Protocol of a simplified and integrated strategy in Burkina Faso and The Gambia: Protocol for the phase 1 of the TRI-MOM project
Source: PLoS One. 2026 Feb 6;21(2):e0322670. doi: 10.1371/journal.pone.0322670 (PMC12880678; doi:10.1371/journal.pone.0322670)

## Slide 1
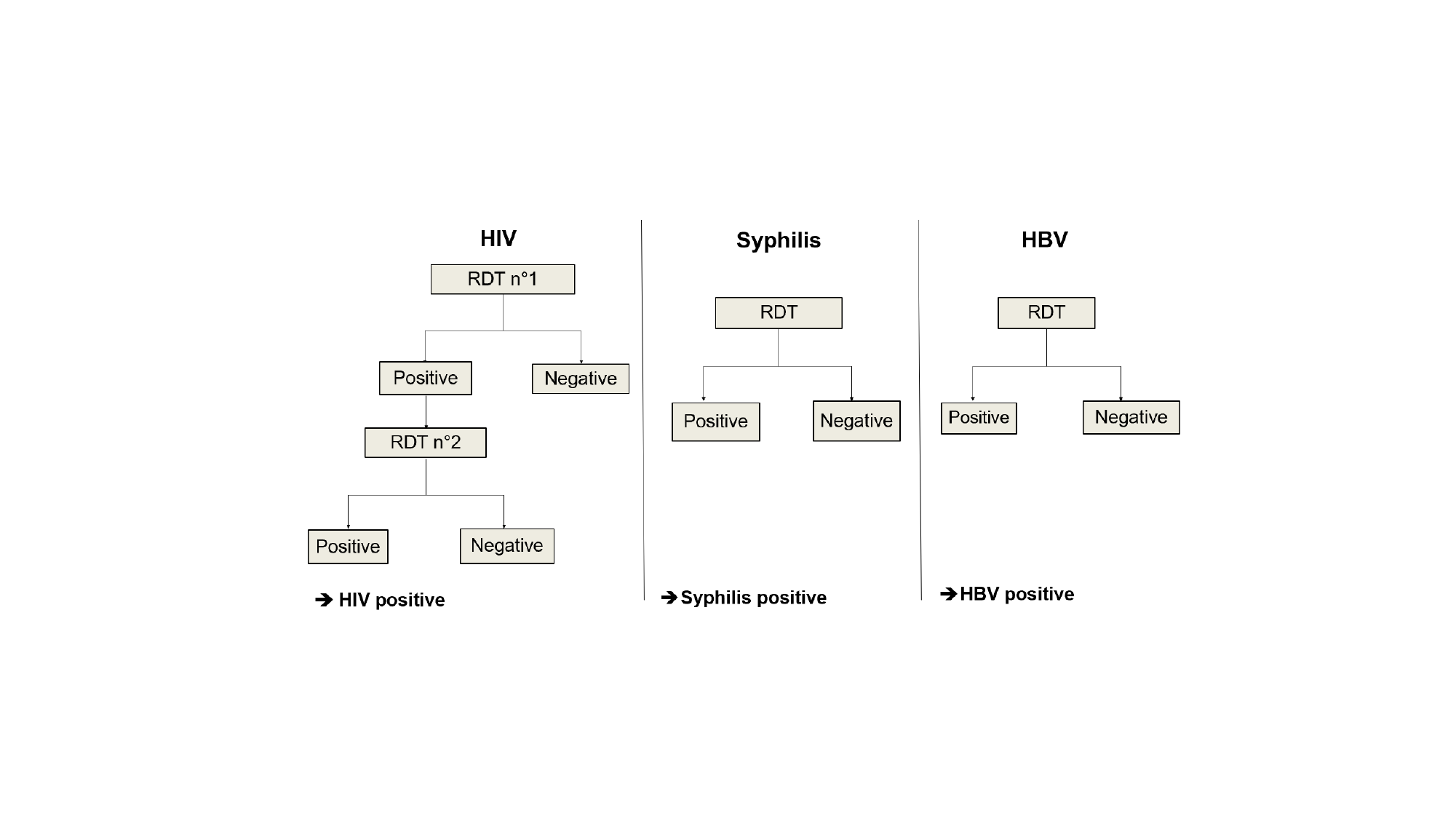

Supplement: S1 Fig — This figure presents the diagnostic algorithm used for prenatal screening of HIV, syphilis, and hepatitis B virus (HBV), including initial testing and subsequent confirmatory steps. Note: RDT = rapid diagnostic test. In case of any ambiguous RDT result, the test should be repeated. (PPTX) [file pone.0322670.s001.pptx]
