# Supplementary material for: Towards triple elimination of HIV, syphilis and HBV mother-to-child transmission: Protocol of a simplified and integrated strategy in Burkina Faso and The Gambia: Protocol for the phase 1 of the TRI-MOM project
Source: PLoS One. 2026 Feb 6;21(2):e0322670. doi: 10.1371/journal.pone.0322670 (PMC12880678; doi:10.1371/journal.pone.0322670)
Supplement: Attachment — Submitted filename: 20251105_response_letter.docx [file pone.0322670.s003.docx]

We thank the editors for their review, and we respond point by point to the questions.

2. We note that the grant information you provided in the ‘Funding Information’ and ‘Financial Disclosure’ sections do not match.

When you resubmit, please ensure that you provide the correct grant numbers for the awards you received for your study in the ‘Funding Information’ section.

We checked the information provided in both Funding Information’ and ‘Financial Disclosure’ sections.

3. Thank you for stating the following in the Competing Interests section:

"I have read the journal's policy and the authors of this manuscript have the following competing interests: GN, and YS report research funding and consultancy fees from Gilead Sciences. ML reports consultancy fees from Abbott USA and Gilead Sciences. All other authors declare no competing interests."

We added this in the competing interests section.

Please confirm that this does not alter your adherence to all PLOS ONE policies on sharing data and materials, by including the following statement: "This does not alter our adherence to  PLOS ONE policies on sharing data and materials.” (as detailed online in our guide for authors <http://journals.plos.org/plosone/s/competing-interests>).  If there are restrictions on sharing of data and/or materials, please state these. Please note that we cannot proceed with consideration of your article until this information has been declared.

Please include your updated Competing Interests statement in your cover letter; we will change the online submission form on your behalf.

We added the sentence into the text.

4. We note that the original protocol that you have uploaded as a Supporting Information file contains an institutional logo. As this logo is likely copyrighted, we ask that you please remove it from this file and upload an updated version upon resubmission.

We removed it from the file and uploaded an update version.

5. Please provide a complete Data Availability Statement in the submission form, ensuring you include all necessary access information or a reason for why you are unable to make your data freely accessible. If your research concerns only data provided within your submission, please write "All data are in the manuscript and/or supporting information files" as your Data Availability Statement.

As requested, we added a section about data availability statement.

6. Please ensure that you refer to Figure 3 in your text as, if accepted, production will need this reference to link the reader to the figure.

If the reviewer comments include a recommendation to cite specific previously published works, please review and evaluate these publications to determine whether they are relevant and should be cited. There is no requirement to cite these works unless the editor has indicated otherwise.

We thank the editor, and we added Figure 3 in the main text.

Additional Editor Comments:

The article is relevant and aligns with the WHO’s strategy for the triple elimination of mother-to-child transmission (MTCT) of HIV, syphilis, and hepatitis B. It presents an interesting approach to public policy development in Sub-Saharan African countries. However, the authors should clarify some points in the manuscript.

More detailed information is needed on the MTCT situation in the two countries studied—specifically, the challenges and opportunities, as well as the similarities and differences between them.

It is also important to explain the rationale for presenting aggregated indicators for the three infections, rather than analyzing the outcomes for each infection separately.

Providing more detail about the selected study sites would also help readers better understand the context and the relevance of the findings.

We agree with the editors, and we added MTCT epidemiology as well as challenges in both countries The Gambia and Burkina Faso.

Reviewers' comments:

Reviewer's Responses to Questions

**Comments to the Author**

1. Does the manuscript provide a valid rationale for the proposed study, with clearly identified and justified research questions?

The research question outlined is expected to address a valid academic problem or topic and contribute to the base of knowledge in the field.

Reviewer #1: Partly

Reviewer #2: Yes

2. Is the protocol technically sound and planned in a manner that will lead to a meaningful outcome and allow testing the stated hypotheses?

The manuscript should describe the methods in sufficient detail to prevent undisclosed flexibility in the experimental procedure or analysis pipeline, including sufficient outcome-neutral conditions (e.g. necessary controls, absence of floor or ceiling effects) to test the proposed hypotheses and a statistical power analysis where applicable. As there may be aspects of the methodology and analysis which can only be refined once the work is undertaken, authors should outline potential assumptions and explicitly describe what aspects of the proposed analyses, if any, are exploratory.

Reviewer #1: Partly

Reviewer #2: Yes

3. Is the methodology feasible and described in sufficient detail to allow the work to be replicable?


Descriptions of methods and materials in the protocol should be reported in sufficient detail for another researcher to reproduce all experiments and analyses. The protocol should describe the appropriate controls, sample size calculations, and replication needed to ensure that the data are robust and reproducible.

Reviewer #1: Yes

Reviewer #2: Yes

4. Have the authors described where all data underlying the findings will be made available when the study is complete?

The [PLOS Data policy](https://track.editorialmanager.com/CL0/https:%2F%2Fjournals.plos.org%2Fplosone%2Fs%2Fmaterials-and-software-sharing/1/010f01980dd29f49-b6fca9e0-183e-4570-89f7-628b4be4146b-000000/TFasIJI35XuxjIM-zs7ARcD55-6LfxlPUOlRZDOTELU=219) requires authors to make all data underlying the findings described in their manuscript fully available without restriction, with rare exception, at the time of publication. The data should be provided as part of the manuscript or its supporting information, or deposited to a public repository. For example, in addition to summary statistics, the data points behind means, medians and variance measures should be available. If there are restrictions on publicly sharing data—e.g. participant privacy or use of data from a third party—those must be specified.

Reviewer #1: No

Reviewer #2: Yes

5. Is the manuscript presented in an intelligible fashion and written in standard English?

PLOS ONE does not copyedit accepted manuscripts, so the language in submitted articles must be clear, correct, and unambiguous. Any typographical or grammatical errors should be corrected at revision, so please note any specific errors here.

Reviewer #1: Yes

Reviewer #2: Yes

6. Review Comments to the Author

Please use the space provided to explain your answers to the questions above and, if applicable, provide comments about issues authors must address before this protocol can be accepted for publication. You may also include additional comments for the author, including concerns about research or publication ethics.

You may also provide optional suggestions and comments to authors that they might find helpful in planning their study.

(Please upload your review as an attachment if it exceeds 20,000 characters)

Reviewer #1: The authors present a summary of their protocol for a study to be conducted in Burkina Faso and the Gambia that will implement a model for integrated clinical services to improve prevention of vertical transmission of HIV, congenital syphilis, and hep B. The aim is for the study findings to inform broader efforts in the countries, region, and Africa to achieve triple elimination of these diseases.

The paper is somewhat lacking in its understanding of how their study would fit into broader initiatives to achieve triple elimination. There is some overreach on how their study can inform scale-up, even locally. For example, while reported to include a mix of rural and urban sites, there are no rural sites in Burkina Faso. While this may be appropriate (given the discussion with the Ministry to select the sites), it would be useful to have more local context integrated into the text to explain why the site selection worked out this way.

On the other hand, the actual individual enrollment numbers for the quantitative survey and cohort study are substantial. Additionally, while the inclusion of a variety of study designs is interesting, the depth of description of the mixed methods evaluation is lacking.

Overall, the balance could be shifted more towards the 2 countries and less towards the “big picture” that is contingent on the study findings and experiences with actual implementation

We thank the reviewer for this feedback, which made us enrich the presentation of what our project will add to the existing field of literature, and of the extent to which we expect to generalize our findings.

Title
1. The reference to this being a protocol paper should appear in the title.

We amended the title to explicit that this paper is a protocol.

Former version

Towards triple elimination of HIV, syphilis and HBV mother-to-child transmission: protocol of a simplified and integrated strategy in Burkina Faso and The Gambia: the TRI-MOM project, phase 1

New version

Towards triple elimination of HIV, syphilis and HBV mother-to-child transmission: protocol of a simplified and integrated strategy in Burkina Faso and The Gambia: protocol for the phase 1 of the TRI-MOM project

Abstract
2. Clarify what the cost and CEA will examine.

We expanded the corresponding paragraph of the abstract.

Former version

Methods and analysis: The strategy will be implemented in 10 rural and urban health facilities in both countries including four activities: i) training sessions for healthcare workers working in maternal and child health services, ii) screening of pregnant women of the three infections using rapid diagnostic tests at the first antenatal visit, iii) clinical assessment and treatment of women tested positive for any of the 3 infections, and iv) raising awareness on HIV, Syphilis and HBV PMTCT among pregnant women and empowering those screened positive. 17,000 pregnant women are expected to be screened. The strategy will be evaluated through an interdisciplinary, mixed-methods approach comprising three studies: i) a quantitative and qualitative cross-sectional study conducted both before and after the implementation of the strategy to assess its impact on triple screening coverage in pregnant women; ii) a cohort study of pregnant women positive for any of the three infections to assess the coverage of PMTCT measures; and iii) a cost and cost-effectiveness analysis.

New version
Methods and analysis: The strategy will be implemented in 10 rural and urban health facilities in both countries including four activities: i) training sessions for healthcare workers working in maternal and child health services, ii) screening of pregnant women of the three infections using rapid diagnostic tests at the first antenatal visit, iii) clinical assessment and treatment of women tested positive for any of the 3 infections, and iv) raising awareness on HIV, Syphilis and HBV PMTCT among pregnant women and empowering those screened positive. 17,000 pregnant women are expected to be screened. The strategy will be evaluated through an interdisciplinary, mixed-methods approach comprising three studies: i) a quantitative and qualitative cross-sectional study conducted both before and after the implementation of the strategy to assess its impact on triple screening coverage in pregnant women; ii) a cohort study of pregnant women positive for any of the three infections to assess the coverage of PMTCT measures; and iii) a cost and cost-effectiveness analysis of the project compared to the reference situation in each country, which will rely on a micro costing study to estimate the incremental cost of the strategy per mother/child couple compared with the reference situation in each country, and compare it to the number of avoided infections.

3. The text is unclear in how it specifies the sample size. It will be conducted in 5 sites in each country – not 10 sites in each of both countries.

We amended the test for more clarity.

Former version

The strategy will be implemented in 10 rural and urban health facilities in both countries

New version
The strategy will be implemented in 5 rural and urban health facilities in each country

Strengths and limitations

4. Specify the country contexts.

We amended the test for more clarity.

New bullet point

Despite the existence of rapid screening tests and prophylactic treatments, mother-to-child transmission of HIV, Syphilis and HBV persists in both The Gambia and Burkina Faso because coverage rates of screening for the three infections during pregnancy are low.

5. The timeframe for the second phase is provided as starting in 2025 when phase 1 is being presented in this paper under review in 2025. This is confusing. And will there be another paper for phase 2? Please clarify.

Phases 1 and 2 consist of distinct activities (Phase 1 focusing on the pregnancy and the delivery, Phase 2 starting from post-partum and lasting for 6 months for each baby) but there will be an overlap of them of the field, starting from the delivery of the first included woman. The delay for publication may have induced some confusion in the calendar.

The protocol of Phase 2 is at the core of another article, soon to be submitted.

6. The study site sample of 5 per country, split between rural and urban settings may not necessarily be considered “several” if looked at from within individual categories.

We replaced the corresponding bullet point with the first one on the context of Burkina Faso and The Gambia.

Introduction
7. It is unusual to have this section start with sub-headers.

We removed the first subheaders.

8. Please use person-centered language throughout the entire manuscript and supplemental files. For example, “adolescents infected with HIV” should be adolescents acquiring HIV.

We amended the manuscript following your guidance.

9. The epi data are out of date (e.g., 2018). Please update all metrics where such data are available.

We updated all possible data, when more recent was available.

10. The text emphasizes the general global situation more than the local context in these 2 countries. Please refocus and provide a succinct overview of the triple elimination targets before describing the situation with burden of disease and prevention access and uptake in the 2 countries.

We thank the reviewer for their coment, and improved the manuscript: we shortened general considerations on triple elimination, and expanded on the situation of Burkina Faso and The Gambia.

Former version

Despite the existence of rapid screening tests and prophylactic treatments, mother-to-child transmission of HIV, Syphilis and HBV persists in both The Gambia and Burkina Faso because coverage rates of screening for the three infections during pregnancy are low.

New version

Despite the existence of rapid screening tests and prophylactic treatments, mother-to-child transmission of HIV, Syphilis and HBV persists in both The Gambia and Burkina Faso because coverage rates of screening for the three infections during pregnancy are low.

11. With regards to hep B elimination, the most critical aspects are HBsAg screening of pregnant women and a country’s history of vaccination – whether the 3-dose series or birth dose implementation. Local vaccination practices are not mentioned until the end of the section and then in insufficient detail. For example, birth dose is not described in terms of the targeted and universal approaches referenced in WHO triple elimination validation criteria.

We expanded on these matters and specified the strategy adopted by both countries in terms of infant vaccine (universal in both countries)

12. Overall, the section refers more generally to preventing these 3 infections and is lacking in its awareness of the WHO’s triple elimination strategy.

We restructured the introduction and hope it is more specific and precise on our study countries.

Former version

Despite the existence of rapid screening tests and prophylactic treatments, mother-to-child transmission of HIV, Syphilis and HBV persists in both The Gambia and Burkina Faso because coverage rates of screening for the three infections during pregnancy are low.

New version

Despite the existence of rapid screening tests and prophylactic treatments, mother-to-child transmission of HIV, Syphilis and HBV persists in both The Gambia and Burkina Faso because coverage rates of screening for the three infections during pregnancy are low.

Methods
13. It would be clearer if the hypothesis appeared earlier in the manuscript and was integrated into the objectives.

We moved this subsection in the study objectives section, to have the hypotheses following the objectives

14. It is somewhat confusing to have objectives in one section and then have them repeated or supplemented elsewhere in the methods.

We clarified in the manuscript, that we were detailing to which specific objectives the mixed-method study, and then the cohort, referred to. We hope it is clearer now.

We also added a table to sum up the study objectives and the component(s) of the evaluation they require

15. Was there a sample size consideration for the mixed methods study? This is missing.

Sample considerations were located after the subsection on study outcomes, we moved in study for better clarity and fluidity. We thank the reviewer for this comment which improves the readability of the manuscript.

Former version

-i) a random sample of women who delivered in the past four months and who attended one of the study sites for postnatal or immunization services (n=800 in Burkina Faso and 600 in The Gambia).

ii) all healthcare workers and community staff involved in prenatal care and/or maternal and child health and PMTCT activities in the study sites (n=150).

New version

i) a random sample of women who delivered in the past four months and who attended one of the study sites for postnatal or immunization services (n=800 in Burkina Faso and 600 in The Gambia). The sample size was determined to demonstrate a significant increase (with a power of >95%) in access to screening for all three infections, i.e., an increase of 5% to 80% for screening for all three infections among pregnant women attending their first antenatal clinic. The sample size will also provide sufficient power to study in detail the socio-economic and behavioural factors associated with non-use of screening for the three infections among pregnant women.

ii) all healthcare workers and community staff involved in prenatal care and/or maternal and child health and PMTCT activities in the study sites (n=150). These estimates correspond to an exhaustive sample (all caregivers and community personnel involved in prenatal care and maternal and child care activities are eligible)..

16. How will the mixed methods data be analysed? This is missing.

The analysis plan for the mixed method cross-sectional study is presented in the ““Before” and “after” cross-sectional study” subsection in the Statistical analysis section.

Overall participation in each quantitative survey will be described using participation rates, and a comparison of the main sociodemographic characteristics between participants and non-participants will be conducted to identify potential selection bias. The study population will be described in terms of sociodemographic and economic characteristics. For variables with missing values exceeding 10% multiple imputation method will be employed for covariates, and Heckman-type models will be utilised for outcomes. Participant’s characteristics will be compared between the “before” and “after” surveys, using chi-square tests for categorical variables and Mann-Whitney-Wilcoxon tests for continuous variables. In case of significant differences, inverse-probability weighting will be employed to reduce covariate imbalances between participants in the “after” survey (who benefited from the strategy) and participants in the “before” survey (who did not benefit from the strategy) (33). The primary outcome will be computed for both the “before” and “after” surveys, and a probit regression model will be conducted to identify factors associated with triple screening and more specifically the effect of the strategy. A similar approach will be used to assess factors associated with the secondary outcomes and the effect of the strategy.

For the qualitative survey, the Word files containing transcriptions will be analysed manually (thematic analysis) and/or using the IramuteQ software. Thematic analysis will be conducted using a combined deductive and inductive approach.

17. Suggest focusing the text on what the authors intend to inform by way of health practice and policy in the countries where the study will be conducted. While their future study findings can inform efforts in the region and the sub-continent, this is somewhat beyond the scope of the proposed study. It remains to be seen if and how the study data could/would be directly generalizeable to the “triple elimination agenda in Africa” as a whole.

We thank the reviewer for this important comment. We agree that the primary scope of our project is to implement and evaluate a triple elimination strategy in two countries, across both urban and rural settings. Our main objective is to generate robust evidence on feasibility, acceptability, and cost-effectiveness that can directly inform national programs and policymakers in Burkina Faso and The Gambia.

We also acknowledge that countries in sub-Saharan Africa share several structural and health system characteristics that may render some of our findings informative beyond the study sites. However, we concur with the reviewer that there are also important differences across regions, such as variations in HIV prevalence, HBV vaccination coverage, and health service delivery models, which may limit direct generalisability of our results. Therefore, while our study will provide valuable insights for the broader “triple elimination” agenda in Africa, further implementation research in diverse epidemiological and health system contexts will be required to identify specific barriers and facilitators in other regions, particularly in East and Southern Africa.

We added a sentence in “Expected impact and dissemination plan”.

18. Overall readability is limited. Although a protocol paper, it would be of greater interest if it added more information (through data, narrative explanation) for a reader who wanted to learn more about challenges and opportunities to achieve triple elimination targets in these 2 countries.

We thank the reviewer, and we added sentences in introduction about challenges and opportunities.

Reviewer #2: The article is highly relevant and aligns with Sustainable Development Goal 3, particularly with regard to the elimination of diseases such as vertically transmitted infections (HIV, syphilis, and hepatitis B).

It also proposes the integration of hepatitis B vertical transmission prevention strategies with existing—though not yet well implemented—initiatives aimed at preventing vertical transmission of HIV and syphilis. This is especially pertinent given that public policies addressing hepatitis B in Sub-Saharan African countries remain underdeveloped.

We thank the reviewer for his comment.

The implementation of the project described in the protocol has the potential to strengthen local efforts to eliminate vertical transmission and to enhance the maternal and child healthcare continuum.

The following points should be better explained:

- When will the Antenatal Care Panel test for HIV, syphilis, and hepatitis B surface antigen (HBsAg) (Abbott, USA) be used, and when will the SD BIOLINETM HIV/Syphilis Duo Test and DetermineTM HBsAg test be used?

Here, we used two approaches to test pregnant women for HIV, syphilis, and HBV. First, national programs in both countries use the SD BIOLINE™ HIV/Syphilis Duo Test in antenatal clinics. To ensure HBsAg testing in addition to these two infections, we provided the Determine™ HBsAg test. Second, we implemented the new Antenatal Care Panel test for HIV, syphilis, and HBsAg (Abbott, USA) in The Gambia at one site (Banjulinding). Data on the type of test used were collected at baseline, and we will assess whether test uptake differed according to the testing approach.

- For what purpose, and for how long, will the biobank samples be stored?

The biobank will primarily be used for virological analyses prior to treatment initiation, including HBV DNA, HIV RNA, and additional biomarkers that may predict mother-to-child transmission. It will also enable characterization and comparison of the virological profiles of infected mothers across both countries. At birth, samples will be used to: estimate adherence to AVT (or ART), and assess the association between virological parameters (HBeAg/HBV DNA, HIV RNA) and transmission outcomes. In addition, as part of the PROTECT-B study, the biobank will allow assessment of the correlation between HBV DNA levels and HBcrAg. Specimens will be stored for a minimum of 10 years (to be confirmed by Gibril; current budget may cover 5 years).

It is also recommended to review the outcome indicators, especially when aggregating all infections into a single metric. Consider whether to maintain a composite indicator or to disaggregate outcomes by infection type. This would allow for a better understanding of the specific implementation aspects of the vertical transmission prevention protocol for each infection (e.g., the proportion of women at risk of MTCT who achieved virological suppression [for HIV and HBV infections] or cure [for syphilis] at delivery -Would it be more appropriate to report these infections jointly or separately?).

We thank the reviewer for this comment. The cascade of care for the three infections will be assessed both overall and by infection. A descriptive analysis will be conducted, and we agree to provide overall estimates so that the triple elimination strategy can be evaluated comprehensively. We are about to submit the TRI-MOM phase 2 research protocol, which will follow mothers and infants up to 6 months after delivery. The primary endpoint is the rate of HBV MTCT at 6 months, as our strategy specifically aims to reduce HBV MTCT below the levels previously reported in the literature.

7. PLOS authors have the option to publish the peer review history of their article ([what does this mean?](https://track.editorialmanager.com/CL0/https:%2F%2Fjournals.plos.org%2Fplosone%2Fs%2Feditorial-and-peer-review-process%23loc-peer-review-history/1/010f01980dd29f49-b6fca9e0-183e-4570-89f7-628b4be4146b-000000/5BDSQyL3RWxdHxYrn1LSroBdv0sBa4DDz9FT9ceylHk=219)). If published, this will include your full peer review and any attached files.

If you choose “no”, your identity will remain anonymous but your review may still be made public.

**Do you want your identity to be public for this peer review?** For information about this choice, including consent withdrawal, please see our [Privacy Policy](https://track.editorialmanager.com/CL0/https:%2F%2Fwww.plos.org%2Fprivacy-policy/1/010f01980dd29f49-b6fca9e0-183e-4570-89f7-628b4be4146b-000000/cLIO7IQtbyIIhnxE2fi2S3dxZxwuW3ohAIOfghSt5Qc=219).

Reviewer #1: No

Reviewer #2: No

[NOTE: If reviewer comments were submitted as an attachment file, they will be attached to this email and accessible via the submission site. Please log into your account, locate the manuscript record, and check for the action link "View Attachments". If this link does not appear, there are no attachment files.]

While revising your submission, please upload your figure files to the Preflight Analysis and Conversion Engine (PACE) digital diagnostic tool, [https://pacev2.apexcovantage.com/](https://track.editorialmanager.com/CL0/https:%2F%2Fpacev2.apexcovantage.com%2F/1/010f01980dd29f49-b6fca9e0-183e-4570-89f7-628b4be4146b-000000/wQ_6gpkXxNiRBdw-EG0bNrGVzhj903qiDzsRVxSH6As=219). PACE helps ensure that figures meet PLOS requirements. To use PACE, you must first register as a user. Registration is free. Then, login and navigate to the UPLOAD tab, where you will find detailed instructions on how to use the tool. If you encounter any issues or have any questions when using PACE, please email PLOS at [figures@plos.org](mailto:figures@plos.org). Please note that Supporting Information files do not need this step.
